# Process evaluation of the implementation of the assessment of burden of chronic conditions tool in Dutch primary care – lessons from a qualitative implementation study

**DOI:** 10.1186/s12913-024-11270-y

**Published:** 2024-07-20

**Authors:** Danny Claessens, Marcia Vervloet, Esther A. Boudewijns, Lotte C.E.M. Keijsers, Annerika H.M. Gidding-Slok, Onno C.P. van Schayck, Bjorn Winkens, Liset van Dijk

**Affiliations:** 1 Department of Family Medicine, Care and Public Health Research Institute (CAPHRI), Maastricht University, P.O. Box 616, Maastricht, 6200 MD the Netherlands; 2 Nivel, Netherlands Institute for Health Services Research, Utrecht, the Netherlands; 3 Department of Methodology and Statistics, Care and Public Health Research Institute (CAPHRI), Maastricht University, Maastricht, the Netherlands; 4 Department of Pharmacotherapy, -Epidemiology and -Economics, Groningen Research Institute of Pharmacy, Faculty of Science and Engineering, University of Groningen, Groningen, the Netherlands

**Keywords:** Process-evaluation, Implementation science, Fidelity, Barriers, Facilitators, Chronic care, Primary care, Qualitative interview

## Abstract

**Background:**

The Assessment of Burden of Chronic Conditions (ABCC-)tool is developed to facilitate a personalized approach to care in the patient-healthcare provider (HCP) conversation based on shared decision-making and individualized care plans. An effectiveness study highlighted its effect on the perceived quality of care and patient activation. Successful implementation of novel interventions necessitates an understanding of the user’s actual application, user experiences and an evaluation of implementation outcomes. This study aims to evaluate the implementation of the ABCC-tool by HCPs in Dutch primary care.

**Methods:**

This study is the process evaluation of a larger type 1 effectiveness-implementation hybrid trial. Semi-structured interviews with HCPs, who were interventionists in the hybrid trial, were held at three and twelve months after they started using the ABCC-tool. The Reach-Effectiveness-Adoption-Implementation-Maintenance (RE-AIM) framework was used to evaluate implementation outcomes. The Implementation domain was further strengthened with an evaluation of implementation fidelity using Carroll’s framework. Inductive coding and thematic analysis were applied to identify relevant participant experiences and implementation outcomes within the RE-AIM framework.

**Results:**

Seventeen HCPs (1 general practitioner, 16 practice nurses) participated in the study, representing 39% of potentially eligible participants. Most HCPs applied the tool after finishing their own routines instead of how it is intended to be used, namely from the beginning of the consultation. HCPs reached 2–6 patients. The ABCC-tool was initially adopted, but twelve HCPs stopped using the tool due to COVID-19 related cancellation of consultations. High fidelity was found for applying the questionnaire and visualization. Low fidelity was present for applying shared decision-making, formulating care goals and monitoring progress. HCPs indicated that maintaning the ABCC-tool depended on accompanying training and implementation support.

**Conclusions:**

HCPs applied the ABCC-tool critically different from intended, potentially diminishing its benefits and ease of use. This evaluation stresses the need for a tailored implementation plan that includes more detailed training and guidance on how and when to use the ABCC-tool.

**Supplementary Information:**

The online version contains supplementary material available at 10.1186/s12913-024-11270-y.

## Contributions to the literature

Fidelity is an important mediator of an intervention’s perceived benefit and implementation success. This evaluation stresses the need for clear training and guidance on using the ABCC-tool.

This type 1 effectiveness-implementation hybrid trial confirms the essential role of a thorough implementation plan when implementing the ABCC-tool in Dutch primary care.

Implementation plans should include tailored strategies based on contextual influences and local needs of implementers.

**Supplementary Information:**

The online version contains supplementary material available at 10.1186/s12913-024-11270-y.

## Background

**Fig. 1:**
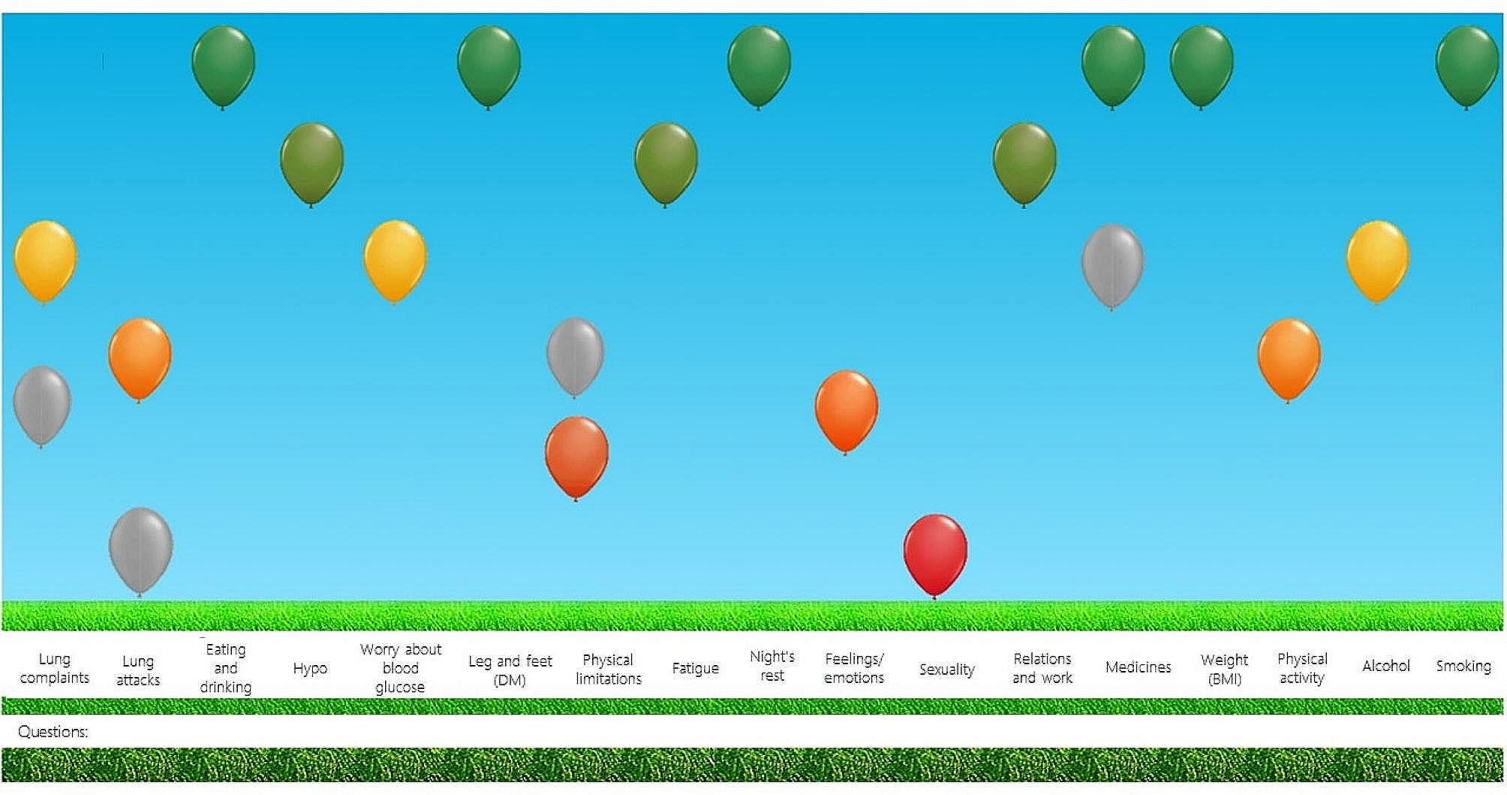
Example of the visualization of the ABCC-tool’s outcomes. Each balloon represents a unique aspect of burden of disease, with high and green balloons representing low burden, red and low balloons representing high burden. Grey balloons represent the last visit’s results. Additional information or questions can be communicated through the open text field. Abbreviations: ABCC – Assessment of Burden of Chronic Conditions, DM – Diabetes Mellitus, BMI – Body Mass Index

The Assessment of Burden of Chronic Conditions (ABCC-)tool has been developed to provide a structured way to deliver personalized chronic care that targets the most pressing needs of the patient [1]. The ABCC-tool assesses and visualizes the patient’s experienced burden of disease (Fig. 1), incorporates this into the patient-healthcare provider (HCP) conversation, and facilitates the formulation of personalized care plans through shared decision-making [1]. However, for HCPs and patients to benefit from the ABCC-tool’s potential, it needs to be adopted and used to reach high fidelity in daily practice. The implementation of interventions such as the ABCC-tool in Dutch general practices is challenging and rarely described due to the scattered nature of Dutch general practices (i.e. general practices operate individually with some local collaboration, but without shared management).

The development of novel interventions in healthcare normally targets the improvement of health status or health delivery, but many interventions fail to reach real-world impact. Implementation studies aim to understand and guide the implementation of novel interventions. Implementation of novel interventions is a rigorous effort that necessitates an understanding of the intervention itself, the users and their context, and the actual implementation of the intervention in real-world settings [2–5]. Interventions are deployed by users and this deployment is influenced by characteristics of the intervention itself, users’ personal skills and values, and their direct and indirect surroundings (e.g. barriers and facilitators) [6]. Even more, the personal experience of users shape the intervention’s success or failure to achieve change [7].

After the development and validation of the ABCC-tool, a type 1 effectiveness-implementation hybrid trial was designed to evaluate effectiveness, implementation context, actual implementation and user experiences [1, 8–10]. The effectiveness study highlighted the ABCC-tool’s effectiveness with regard to patients’ experienced quality of care and patient activation [11]. A context-analysis evaluated HCP’s expected barriers and facilitators to the implementation of the ABCC-tool in Dutch general practices, highlighting that an implementation plan should incorporate strategies that target both the inner and the outer setting of general practices [12]. The current study aims to describe HCPs’ implementation process, outcomes and experiences in implementing the ABCC-tool during a type 1 effectiveness-implementation hybrid study, and to further inform a targeted implementation plan for successful large-scale implementation of the ABCC-tool in Dutch primary care.

## Methods

### Study design

A longitudinal qualitative process evaluation was performed amongst HCPs that participated as interventionists in a larger type 1 effectiveness-implementation hybrid study, of which the protocol was described elsewhere [10]. In short, an effectiveness study among patients was combined with a context evaluation and process evaluation among the HCPs that were using the ABCC-tool as an intervention in their practice. HCPs that used the ABCC-tool were interviewed at 3 and 12 months follow-up to evaluate their user experiences and to assess implementation outcomes. The study lasted from December 2019 until August 2022. All interviews were performed via video-conferencing. The reporting of this implementation study was guided by the Standards for Reporting Implementation Studies (see additional file 1).

### Population and recruitment

Participating HCPs were interventionists in the parallel effectiveness trial among patients. HCPs were informed about the opportunity to be included in the implementation study after inclusion in the effectiveness-part of the larger trial. Recruitment of participants was assisted by the ZIO care group (explained below). As such, this study used a convenience sample. As detailed in the protocol, a minimum of 15 participants was estimated to observe data saturation with regard to user experiences [10].

### Context of Dutch primary care

The outcomes of this study need to be interpreted in the context of Dutch primary care. In short, primary care in the Netherlands is accessible to all residents through mandatory health insurance, and is the first line of care. Medical care is provided by General Practitioners (GPs) or delegated to practice nurses (PNs) or nurse practitioners (NPs) when concerning chronic care. Because both PNs and NPs perform the same type of care with regard to using the ABCC-tool, no distinction will be made and all will be indicated as PN. GPs and PNs are often organized within care groups, which are legal bodies that organize healthcare through negotiating payments from insurers, providing information systems or employ PNs [13]. Integrated care programs structure this delegated care and facilitate workflows and protocols according to which PNs provide healthcare. GPs and PNs register care in their own digital information systems (GP information systems or integrated care information systems). Additional digital services (such as patient platforms and eHealth) are provided as part of integrated care information systems or by third party providers. Primary care is strongly guided by the Dutch College of General Practitioners through care standards and guidelines. A detailed context-analysis has been published elsewhere, describing relevant contextual influences for the implementation of the ABCC-tool [12].

### The intervention: the ABCC-tool

The ABCC-tool has been developed as a structured instrument to guide the conversation between HCPs and people with chronic conditions towards personalized care plans. The tool aims to assess a person’s experienced burden using a questionnaire, visualize this burden during the conversation (see Fig. 1) and facilitate the formulation of personalized care plans through burden-specific treatment advice. It consists of a generic module which is always combined with disease specific modules (at the moment available for Asthma, Chronic Obstructive Pulmonary Disorder (COPD), Type 2 Diabetes Mellitus (T2DM) and Chronic Heart Failure (CHF)). The tool is specifically intended to be used at the start of the clinical consultation, and as such the questionnaire would have been completed beforehand. HCPs use the ABCC-tool in a 5-step cycle, being: (1) assess burden (i.e. complete a questionnaire), (2) visualize its results, (3) discuss a selection of topics that are relevant to the patient through means of shared decision-making, (4) translate health issues into personalized care goals, and (5) monitor the progress of experienced burden and care goals in subsequent visits. The ABCC-tool, including its core (questionnaire, visualization, treatment advice) and adaptable (patient preparation, applying shared decision-making, formulate care goals and plans, and monitor patient progress) components and intended use, is described in more detail in additional file 2.

### Implementation strategies

A limited selection of non-targeted implementation strategies was applied to enable HCPs to use the ABCC-tool. The ABCC-tool is incorporated into HCP’s own digital systems (through third party digital systems) and HCPs were provided with practical information about using the ABCC-tool in paper and an instruction video (detailed in additional file 2). Additionally, HCPs using the integrated care information system received technical support (i.e. remote assistance in case of information system errors of malfunction that would compromise using the ABCC-tool) and recruitment support (i.e. telephone calls to remind HCPs to recruit patients, to evaluate recruitment progress and advice to change recruitment strategies from passive to active recruitment) from their care group. Alterations to the implementation strategies were not allowed during the course of this study to minimize their impact on the co-occurring effectiveness trial among patients. A detailed description was provided elsewhere [10].

### Outcomes

The primary outcomes of this study were the implementation outcomes as described by the Reach-Effectiveness-Adoption-Implementation-Maintenance (RE-AIM) framework [10, 14–16]. Additionally, user experiences, including experienced benefits and drawbacks, were evaluated with regard to using and implementing the ABCC-tool in daily practice. Even more, participant characteristics were collected regarding their age (in years), occupation (GP, PN), years of practical experience, prior experience with the tool’s predecessor (because of high resemblance in the conversation approach) and number of patients participating. Due to COVID-19, many participants and their patients stopped using the ABCC-tool. Thus, it was not possible to complete the planned follow-up of barriers over time. Table 1 presents an overview of the implementation outcomes and their operationalization.

**Table 1:** Implementation outcomes as derived from the Reach-Effectiveness-Adoption-Implementation-Maintenance (RE-AIM) framework

Outcome	Operationalization
Reach	Number of patients * Reflection on participant characteristics with regard to representativeness or suitability for the ABCC-tool
Effectiveness	Personal experience of the effect of the ABCC-tool on a HCP’s practice or knowledge **
Adoption	Continued use of the ABCC-tool during trial
Implementation	Actual use of ABCC-tool Fidelity (additional file 2) Experience with using the tool
Maintenance	Intent and actual preparation for maintaining use in practice after study end

Table 1. The in-depth evaluation of intervention fidelity is based on the framework by Carroll et al. [17]. * HCPs were instructed to include 5–10 patients for the effectiveness trial. ** Actual effectiveness was evaluated in an effectiveness trial. Abbreviations: HCP = healthcare provider, ABCC-tool = Assessment of Burden of Chronic Conditions tool

### Data analysis

All interviews were audio-recorded, transcribed verbatim at literatim and pseudonymised. Due to the many similarities between the 3-months and 12-months interviews with regard to user experiences and the fact that a large proportion of participants had dropped-out before the 12-month interview, these were analyzed together. All interviews were processed using inductive coding within the broad structure of the RE-AIM framework [14–16]. Implementation fidelity was processed using inductive coding within the various steps of the process of using the ABCC-tool [10, 17]. A thematic analysis was performed to identify the relevant themes describing each of the domains of the RE-AIM framework and Carroll’s implementation fidelity framework, and to describe the user experiences of participants. To minimize the risk of researcher bias, three out of seventeen interviews were randomly selected and independently coded and discussed by two researchers (DC, MV) and upon consensus coding was continued by one researcher (DC). All coding and analyses were performed using Nvivo (NVivo qualitative software (version 12) [computer software]. Lumiverio).

## Results

### Participants

Seventeen HCPs, of the 44 HCPs in the larger hybrid trial, participated in this implementation study (being 39% of eligible participants). Thirteen of them accessed the ABCC-tool through the GP information system and four made use of an integrated care information system. One of the participants was a GP and only used the ABCC-tool through multidisciplinary discussions with a participating PN from the same practice. The remaining 16 participants were either a nurse practitioner or practice nurse. Participant characteristics are presented in Table 2. Three-month and twelve-month interviews had a median duration of 25 min (range 14–45) and 39 min (range 26–61) respectively. Five participants completed the study (i.e. used the ABCC-tool until the 12-month interview). The remaining participants had stopped using the ABCC-tool before the 3-month interview (*n* = 3), right after the 3-month interview (*n* = 4), or somewhere in between the 3-month and 12-month interview (*n* = 5). Seven of the twelve HCPs that stopped using the ABCC-tool did so during periods of COVID-19 induced national lockdowns.

**Table 2:** Characteristics of participants and their general practices

Individual characteristics	
Age, years	
- Median (range)	51 (30–65)
Occupation, *n*	
- Practice nurse - Nurse practitioner - General practitioner	13 3 1
Years of experience, years	
- Median (range)	11 (0–20)
Prior experience tool’s predecessor*, n	
- Practical experience - Followed training - Provided training	9 8 2
Number of patient participants	
- Median (range)	3 (0–6)

Table 2 describes the characteristics of participants. * the Assessment of Burden of COPD (ABC) tool [31]. Abbreviations: ABCC = Assessment of Burden of Chronic Conditions, n = number

### Implementation outcomes

#### Reach

HCPs stated that they included two to six patients, from the requested five to ten, with whom they expected easy use. In contrast to their population having limited self-management skills and disease awareness, HCPs invited patients who had high self-management skills and disease awareness as well as were relatively younger and well educated. Some HCPs added that the included patients may not have been suited for using the ABCC-tool because they had little to gain from the tool in the first place. Additionally, HCPs stated that the ABCC-tool may require modification (e.g. simplification in language and number of possible answers) to be compatible with patients who have lower (health) literacy and lower socio-economic status. HCPs explained that most of their patients had stopped using the tool during the trial due to the COVID-19 pandemic. Other reasons for stopping the use of the tool were completing questionnaires for study purposes alongside the questionnaire that was part of the ABCC-tool (i.e. high participant burden for patients as experienced by HCPs), and patients not experiencing any benefit from the ABCC-tool.

*“So they already had a lot of knowledge and little burden…. So no, I would not keep using the tool with these people” – HCP 13.2*.

#### Experience of effectiveness of the intervention

The results of the effectiveness trial are published separately [11]. HCPs explained that the ABCC-tool expanded their knowledge of the experience of disease of their patients, enabled HCPs to delve into behaviors their patients were actually willing to change, and increased their awareness on the possibilities for patient self-management.

*“Using the ABCC-tool, I became more aware of what patients could to themselves” – HCP 19.2*.

#### Adoption

##### Actual adoption

All HCPs explained that the ABCC-tool should primarily be adopted by PNs, though some PNs proposed that using the ABCC-tool should not be an individual task but a team approach where each team member (e.g. GP, PN) uses the ABCC-tool when appropriate and useful for the patient. All HCPs explained that using the ABCC-tool had diminished over time, mainly due to COVID-19-related reasons. HCPs explained using the ABCC-tool became less of a priority.

*“Well I was quite enthusiastic in the beginning… And then it just faded away, from my end and from the patient’s” – HCP 30*.

##### Influences to the adoption process

HCPs elaborated on reasons they stopped using the ABCC-tool. Some HCPs explained that during COVID-19 lockdowns, patients lost overall motivation to come to consultations and use the ABCC-tool. HCPs explained that this was a reason to stop using the ABCC-tool altogether.

*“Well since we last spoke I haven’t [used the ABCC-tool] anymore. Just because so much effort went into reaching patients, and they just cancelled everything” – HCP 30*.

HCPs noticed several factors that impacted the adoption of the ABCC-tool from the HCPs perspective. Additionally, most HCPs mentioned that they felt that using the ABCC-tool in their selection of patients cost too much time for the limited benefits they experienced. High patient demand, a shift in priorities (e.g. because of separation of a practice into two practices), or patients transferring to a colleague, were additional reasons for a PN to stop using the ABCC-tool.

All HCPs acknowledged that they had to change their conversation routine in order to use the ABCC-tool, as they now should start the conversation with the ABCC-tool.

*“I mean, we are still in our same old routines, so we first still have the old conversation… and then, it does not save you any time at all” – HCP 35*.

Additionally, preparing a consultation by sending questionnaires to patients was experienced as incompatible with current workflows. Technical issues had a major influence on the adoption. The multi-step access and inability to automatically transfer data (e.g. such as ABCC-tool visualizations or formulated care goals) between digital systems complicated the use of the ABCC-tool.

*“And this is yet another system, you know? It would be ideal to have it all in one and the same window. Now it is too many systems” – HCP 30*.

*“It keeps coming back to this issue of having to keep copying information. And that costs so much time. So if that could be fixed, that would help a lot.” – HCP 19.1*.

HCPs generally felt short of time in regular care and some HCPs experienced that implementing the ABCC-tool was not feasible in this situation.

#### Implementation fidelity and experiences

##### Timing of the tool within the consultation

Most HCPs described an essential and major deviation from the instructed use of the ABCC-tool, namely that they applied the ABCC-tool only after finishing their own routine conversation with their patients instead of starting the conversation with the ABCC-tool. These HCPs therefore experienced the ABCC-tool to be a duplicate of what they had already done and thus a major intrusion of their available time. Only one HCP applied the ABCC-tool from the start of the conversation and commented that this approach did not cost any additional time.

*“I should have said, you completed the ABCC-tool, let’s have a look. But I first asked what I always asked, and then … the ABCC-tool. But then that was a repeat of what we just discussed” – HCP 35*.

##### The ABCC-scale (questionnaire)

All HCPs used the questionnaire of the ABCC-tool during all consultations with included patients. Patients of most HCPs completed the questionnaire at home. Most HCPs provided the questionnaire during the consultation (i.e. for the next consultation), while some sent it via e-mail or postal services. Both options worked to satisfaction for HCPs. Sending digital questionnaires via e-mail allowed HCPs to prepare the ABCC-tool beforehand, leaving them more discussion time during the consultation. When completing questionnaires in the waiting room, HCPs stated that asking patients to arrive 10 min early was acceptable and feasible to HCP, patient, and practice.

*“I asked people to come in early so they had some time to complete the questionnaire [in the waiting room]. They didn’t mind” – HCP 13.2*.

One HCP completed the questionnaire during the consultation, but stated that this method resulted in patients not having enough time to reflect on their experienced burden and in patients providing socially desirable answers.

##### Visualizing the outcomes

Upon completing the questionnaire, all HCPs visualized the outcomes into the balloon chart. All HCPs observed the visualization together with their patients. All HCPs expressed the visualization to be the most useful and innovative aspect of the tool. The balloons were clear, activated patients to discuss their experienced burden, and matched the patient’s experience. Additionally, the visualization reflected the change in health status as experienced by the patient (e.g. worsening of complaints was detected by the ABCC-tool).

*“So this patient worsened and struggled with [his/her] medication. And you could see the difference in the ABCC-tool as well” – HCP 38*.

While HCPs acknowledged the fact that the visualization matched their patients’ experiences, it did not provide new information. Both red and green balloons were equally informative. HCPs experienced presenting the patient with too many red balloons as demotivating to the patient.

*“It can also be positive, right? So if grey balloons arose, or it had green balloons, I would also address those” – HCP 38*.

##### Shared decision-making conversation

Most HCPs explained that they invited their patients to identify relevant topics for discussion. Some HCPs explained that their patients could not be persuaded into active participation and that they had to maintain a leading role in the conversation.

*“I always try to activate people to participate, because otherwise [the consultation] has no point. But people rarely take action” – HCP 92*.

Most HCPs mainly focused on high burden of disease (red balloons). Some HCPs also discussed domains that had a low burden of disease (green balloons). With regard to shared decision-making, some HCPs explained that they provided options for their patients and asked patients about their preferences. In most cases, the effort of the HCP resulted in patients identifying domains that were relevant to them. Most of these HCPs, however, commented that it took much effort to get patient to actively participate. Most HCPs explained that their conversations were more elaborate when compared to not using the ABCC-tool. Some HCPs added that the ABCC-tool facilitated the HCP to get to know their patients better.

##### Formulating personalized care goals

Some HCPs registered care goals. The majority of these goals were of global nature and mostly concerned a broad statement (e.g. maintenance of current status, stop smoking, start exercising). Sometimes HCPs registered more specific goals, but none committed to guidelines with regard to formulating goals (such as the SMART principle) while being familiar with them.

*“It is quite difficult to really make a smart goal. And we do that too little” – HCP 13.1*.

HCPs who registered goals indicated that they considered care goals as a prime focus of their conversation. Most HCPs did, however, not register personal care goals during their consultations. Reasons were patients being satisfied with their current status, and patients not being used to think in terms of care goals.

*“Not all patients are eager or even willing to create goals. Most patients are satisfied the way they are” – HCP 92*.

##### Monitoring progress

Monitoring of patient progress was mostly performed verbally, without the use of the ABCC-tool. Most HCPs referred to the last conversation and informed about any changes since. When goals were formulated, monitoring was performed by asking about the progress with regard to that goal. When goals were not met, they were re-evaluated and changed if necessary.

#### Maintenance

Four HCPs were still working with the ABCC-tool at the time of the final evaluation. One HCP had already prepared colleagues to use the ABCC-tool with all of their new patients. Another HCP is going to re-evaluate using the ABCC-tool with her care group, in order to improve its access and use. However, most HCPs were not using the ABCC-tool anymore at the time of the final evaluation. Most of them expressed a desire to continue using the ABCC-tool, but only after certain improvements or alterations were made (detailed explanation below). One HCP concluded not to work with the ABCC-tool anymore because of her experience of the amount of time it required and not seeing enough benefit for her practice. Most HCPs would recommend the ABCC-tool to provide chronic care, if modifications to the implementation strategies and the tool itself were implemented.

### Implementation experiences

#### Barriers and facilitators to the implementation process

##### The COVID-19 pandemic

This study coincided with the onset of the COVID-19 pandemic. As chronic care was largely postponed, the study was paused until HCPs were having physical consultations again. Even after initial lockdowns, many patients cancelled due to a fear of contracting COVID-19 or them not seeing the benefit of physical consultations anymore. Most consultations were performed via telephone and HCPs explained that using the ABCC-tool was not feasible this way. From the HCPs perspective, the COVID-19 pandemic resulted in alternating high work pressure and postponement of regular care, insecurity about their own safety, and an overall experience of chaotic work circumstances. All deviations were more pronounced in people with pulmonary conditions, and most experiences therefore stem from care for people with T2DM. Overall all HCPs concluded that the COVID-19 pandemic and its aftermath were unsuitable periods to implement new interventions in healthcare practice.

*“It just has not been a good period to try out new things very actively” – HCP 38*.

##### Regular contextual influences on the implementation process

HCPs explained that implementing the ABCC-tool was influenced by several other factors. Firstly, HCPs experienced that the healthcare system expects from them that they see as many patients in a day as is possible. This results in an overall experience of having too little time to be able to deliver care besides the minimum requirements.

*“And it keeps coming back to quickly see people. And then you have to add this to it, and it is just too much” – HCP 30*.

Secondly, HCPs indicated that patients are not used to taking an active role in their own care process, either because of old habits or because they do not want to change an unhealthy lifestyle.

*“… it should be driven by the patient wanting to consult me and wanting to put an effort in [their health]. But that does not happen” – HCP 38*.

Thirdly, most HCPs indicated that too much digital systems are required during the provision of care, and that the ABCC-tool added to this. This multitude of digital systems in which PNs have to work was experienced as complex and unnecessary. Fourthly the study design implied that patients had to complete two different questionnaires (i.e. a research questionnaire to study effectiveness, and the ABCC-questionnaire to assess burden). HCPs expressed that patients felt over-asked and confused by this set-up, which resulted in patients forgetting the ABCC-questionnaire or stopping because of the high participation burden, which in turn influenced the HCP’s motivation to continue using the ABCC-tool.

#### Experienced benefits and drawbacks of the ABCC-tool in practice

HCPs experienced several benefits of using the ABCC-tool. Firstly, the benefit most HCPs mentioned was the overall higher quality of the conversation between HCP and patient, mainly because of clear discussion topics and facilitating the creation of personal care goals. Even more, the ABCC-tool facilitated having the conversation about a patient’s own potential to change their health in general.

“*I think it is a useful aid to really get a grip on the experience of a patient” – HCP 13.1*.

Secondly, most HCPs expressed that the ABCC-tool increased the HCP’s and patient’s insight in the broad experience of a chronic condition and the discussion of topics that are not commonly addressed (e.g. sexuality). Thirdly, some HCPs experienced their patients to be better prepared for the consultation through prior reflection at home and receiving a reminder of the consultation date.

*“They became more pro-active, and had thought about their experiences beforehand. And they were reminded of the appointment they had, which was nice” – HCP 30*.

HCPs also experienced several drawbacks of the ABCC-tool. Firstly, HCPs expressed that using the ABCC-tool as they currently had took too much time. Copying paper questionnaires’ results or registering consultation dates in different digital systems was experienced as too time-consuming and infeasible to current practice. HCPs expected that the time-demand of the ABCC-tool would reduce over time as HCPs would get used to the tool, but they felt like they never got used to the ABCC-tool due to low participant numbers and infrequent visits.

*“It kind of keeps me from using the tool, all the paperwork and administration. It makes the tool a burden to me” – HCP 19.1*.

Secondly, HCPs explained that they already have to complete several patient-reported outcome measures (PROMs), such as the Clinical COPD Questionnaire (CCQ) and the Asthma Control Questionnaire (ACQ), as part of regular care. HCPs added that there is no room for additional questionnaires during the consultation time, and that they are in need for a way to combine these questionnaires into a single measure. Thirdly, some HCPs mentioned the invariant repetitiveness of the ABCC-tool to be a drawback. Patients may get used to the questions and complete them based on habit instead of actually reflecting on the question.

## Discussion

The results of this process evaluation highlight that HCPs applied the ABCC-tool critically different from intended, resulting in time shortages and loss of benefit or effectiveness of the tool, and a premature stop in the adoption process. Implementation was furthermore influenced by HCPs experiencing little time to change their routines, digital information systems which were too complex and scattered, and patients who may not have been empowered to uptake an active role with regard to their own care goals. Several implementation outcomes were evaluated in this process evaluation. Reach of patients by HCPs was initially as expected within study boundaries, but most HCPs and their patients had stopped due to the COVID-19 pandemic. HCPs reached mostly patients with whom they expected easy use, but added that these patients were less suitable for the ABCC-tool’s benefits as they had little to gain with regard to their health status or self-management skills. This indicates the difficulty with selecting adequate patients for the ABCC-tool. The ABCC-tool increased the HCP’s awareness of the patient’s experience and willingness to achieve change. The HCPs’ adoption of the ABCC-tool diminished over time due to the barriers of requiring a behavioral change in patients and HCPs, infeasibility due to limited time availability, and intervention complexity. The questionnaire and visualization were applied with high fidelity, but HCPs found that additional support or training was required to facilitate shared decision-making, formulation of personal care goals and monitoring of patients. The evaluation of implementation outcomes and experiences stresses the need for the development of a thorough and tailored implementation plan which at least contains training and guidance in how and when to use the ABCC-tool.

The effectiveness trial highlighted a significant positive effect of the ABCC-tool on theperceived quality of care by patients and patient activation. The apparent distinction between the implementation process and the ABCC-tool’s effectiveness on patient outcomes may be explained by several hypotheses. First, while the effectiveness may seem striking in respect of the limited adoption and fidelity presented in this process evaluation, this may show the potentially hidden benefit of the ABCC-tool when its implementation is strengthened with a thorough implementation plan. Second, this process evaluation concerns HCP’s experiences while the effectiveness was evaluated in patient outcomes. There may be differences between the implementation experiences of HCPs and the effect using the ABCC-tool has on a patient level. Third, while this study obtained data saturation, it is possible that the experiences of these HCPs do not resemble the entire intervention group of the effectiveness study (which totals into 41 practices). If the intervention group had HCPs that did manage to adopt and implement the ABCC-tool to a higher fidelity, their patients may explain the observed effectiveness. Nonetheless, the current observed effectiveness, under the presented implementation circumstances, warrant the exploration of the ABCC-tool’s effects when implemented with high fidelity.

These results are in line with implementation studies in other similar healthcare settings where digital interventions were evaluated. Firstly, the current results match with user experiences from the ABCC-tool’s predecessor with regard to experience of benefits of applying the tool and the drawbacks of technical difficulties [18]. Incorporating the instrument into HCP’s information systems proved not to be sufficient to warrant easy use. Two recent studies identified time availability and time-to-benefit ratio as a barrier, which were also dependent on compatibility with workflows and the limited number of eligible patients [19, 20]. In addition to the ABCC-tool’s predecessor, another study evaluating a digitalized medication adherence tool stressed the importance of a practical approach in protocols for intervention uptake [18, 20]. These studies strengthen the notion that healthcare professionals require dedicated support and guidance on how to use digital interventions while applying the principles of shared decision-making in patient conversations [18, 19]. Another study that evaluated the process of implementing a blended eHealth illness management and recovery program in mental health alongside an effectiveness trial identified that digital access is an important influence to potential non-use or drop-out [21]. Both studies stressed the need for guidance on selecting adequate participants based on their needs, competences and fit with the intervention [19, 21]. The necessity for implementation guidance and support was also stressed in a study evaluating the implementation of personal health records, where patients access and manage medical data, alongside an effectiveness trial [22]. Additionally, a recent overview of pre-identified influences on the implementation of eHealth highlighted that interventions need to be easy to use, adaptable to workflows, and that users require the time to get used to the intervention and receive implementation support [23]. Additionally, empowering patients to adopt an active participatory role has previously been identified as essential to the implementation of eHealth (even when patients are not the primary users) [24, 25].

This study should be interpreted in light of both its strengths and limitations. First, a major strength of this evaluation is the combination of implementation outcomes alongside experiences to understand implementers’ efforts and struggles. This combination allows for a deeper understanding of the implementation process and the requirements for future implementation plans. Similarly, a second strength of this study is the in-depth evaluation of implementation fidelity alongside implementation experiences. A step-by-step description of the actual use of HCPs helps to understand implementation failure (in case of incorrect use) as well as identify opportunities for the improvement of the ABCC-tool. Third, this continued evaluation during the pandemic allows for the identification of crises-dependent influences of implementation. The experiences of HCPs who continued using the ABCC-tool are lessons for the development of implementation plans that include preparation for crisis-situations. Including crises as a determinant of implementation, as has been done in the updated Consolidated Framework for Implementation Research, highlights the importance for studies that describe implementation efforts during crises [26]. A first limitation of this study design is the inability to develop or adjust implementations strategies during an effectiveness study. While it remains questionable whether HCPs would have time to participate in new strategies when implementing the ABCC-tool itself was experienced as too time consuming during the COVID-19 pandemic, the potential was not explored due to this limitation in design. Second, a major limitation of this study is its co-occurrence with the COVID-19 pandemic, which incapacitated all opportunities to implement the ABCC-tool and led to high drop-out of both HCPs (*n* = 13) and their patients. While the study period was paused during the first Dutch national lockdown, many HCPs experienced implementing the ABCC-tool during the aftermath of the lockdown as infeasible. When such crises uproot regular care and routines, HCPs seem to put most effort into restoring their routines instead of allowing a change of routine. Third, the uprooted care processes during the COVID-19 pandemic and high drop-out rate of participants early in the follow-up period disallowed the follow-up of barriers and facilitators with respect to the evaluation of their natural development over time. The nature of presented barriers during the COVID-19 pandemic was incomparable to the previous situation, and the early drop-out did not allow for follow-up of pre-identified barriers and facilitators. Nonetheless, the pre-identified set of barriers and facilitators can now be updated with determinants of implementation during this process evaluation to allow for the development of an implementation plan with broad coverage of both pre- and post-crises situations.

Additional to the primary outcomes, the process evaluation highlighted important aspects for the implementation of the ABCC-tool (and tools of similar nature). First, even though the ABCC-tool largely has the same contents as guidelines recommend, applying the ABCC-tool required a behavioral change in HCPs that the current implementation strategies insufficiently facilitated. Future implementations strategies need to focus on implementing the ABCC-tool as a change in routine instead of a restructuring of current conversations. Even more, while a great amount of effort is spent in facilitating personal care goals, this evaluation highlights that this is still in its infancy in day-to-day practice. Guidance and skill development is required to support HCPs in adopting the practice of formulating personal care goals [27]. Second, where researchers and HCPs share the idea that patients should have a leading part to play in their own chronic care and health in general, patients may not yet be equipped with the mindset and skills to do so [28]. This stresses that the paradigm shift where patients are empowered to lead their own health through healthy lifestyle and active care participation requires support for patients to develop the necessary skills and expectations. Third, more than anything this process evaluation stresses the development of implementation plans tailored to the needs and context of its implementers [29, 30]. While these needs may differ from a case-to-case perspective, an implementation plan for the ABCC-tool should include simplification of the ABCC-tool with regard to digital complexity and access, and the development of targeted implementation strategies based on the outcomes of a context evaluation and the lessons learnt from this process evaluation.

## Conclusion

This process evaluation describes the implementation of the ABCC-tool amidst the COVID-19 pandemic. The evaluation of implementation outcomes highlights the need for a targeted implementation plan which includes more detailed training and guidance in using the ABCC-tool and tailored strategies based on contextual influences and local needs. User experiences additionally stress the critical influence of the COVID-19 pandemic, as well as the need for implementation efforts to include aspects of behavior change among implementers or even a paradigm shift among intervention recipients.

### Electronic supplementary material

Below is the link to the electronic supplementary material.

Supplementary Material 1

Supplementary Material 2

Supplementary Material 3

## Data Availability

The datasets generated during and/or analysed during the current study are available in the DataHub Maastricht repository, through DataVerseNL access: https://dataverse.nl/dataset.xhtml?persistentId=doi:10.34894/2DXOP0, persistent identifier: doi:10.34894/2DXOP0, upon reasonable request.

## Abbreviations

ABCC: Assessment of Burden of Chronic Conditions
HCP: Healthcare Provider
RE-AIM: Reach-Effectiveness-Adoption-Implementation-Maintenance
GP: General Practitioner
PN: Practice nurse
COPD: Chronic Obstructive Pulmonary Disease
T2DM: Type 2 Diabetes Mellitus
CHF: Chronic Heart Failure
COVID-19: Coronavirus disease 2019

## References

[1] Boudewijns EA, Claessens D, van Schayck OCP, Keijsers L, Salome PL, In ‘t Veen J, et al. ABC-tool reinvented: development of a disease-specific ‘Assessment of Burden of Chronic conditions (ABCC)-tool’ for multiple chronic conditions. BMC Fam Pract. 2020;21(1):11. 31931729 10.1186/s12875-019-1075-8 PMC6958572

[2] Bauer MS, Kirchner J. Implementation science: what is it and why should I care? Psychiatry Res. 2020;283:112376. 31036287 10.1016/j.psychres.2019.04.025

[3] Nilsen P. Making sense of implementation theories, models and frameworks. Implement Sci. 2015;10:53. 25895742 10.1186/s13012-015-0242-0 PMC4406164

[4] Luig T, Asselin J, Sharma AM, Campbell-Scherer DL. Understanding implementation of Complex interventions in Primary Care teams. J Am Board Fam Med. 2018;31(3):431–44. 29743226 10.3122/jabfm.2018.03.170273

[5] Ross J, Stevenson F, Lau R, Murray E. Factors that influence the implementation of e-health: a systematic review of systematic reviews (an update). Implement Sci. 2016;11(1):146. 27782832 10.1186/s13012-016-0510-7 PMC5080780

[6] Nilsen P, Bernhardsson S. Context matters in implementation science: a scoping review of determinant frameworks that describe contextual determinants for implementation outcomes. BMC Health Serv Res. 2019;19(1):189. 30909897 10.1186/s12913-019-4015-3 PMC6432749

[7] Swindle T, Rutledge JM, Martin J, Curran GM. Implementation fidelity, attitudes, and influence: a novel approach to classifying implementer behavior. Implement Sci Commun. 2022;3(1):60. 35668517 10.1186/s43058-022-00307-0 PMC9171954

[8] Claessens D, Boudewijns EA, Keijsers L, Gidding-Slok AHM, Winkens B, van Schayck OCP. Validity and reliability of the Assessment of Burden of Chronic conditions Scale in the Netherlands. Ann Fam Med. 2023;21(2):103–11. 36973066 10.1370/afm.2954 PMC10042564

[9] Boudewijns EA, Claessens D, Joore M, Keijsers L, van Schayck OCP, Winkens B, et al. Effectiveness and cost-effectiveness of the Assessment of Burden of Chronic conditions (ABCC) tool in patients with COPD, asthma, diabetes mellitus type 2 and heart failure: protocol for a pragmatic clustered quasi-experimental study. BMJ Open. 2020;10(11):e037693. 33203626 10.1136/bmjopen-2020-037693 PMC7674093

[10] Claessens D, Vervloet M, Boudewijns EA, Keijsers L, Gidding-Slok AHM, van Schayck OCP, et al. Understanding the healthcare providers’ perspective for bringing the assessment of burden of chronic conditions tool to practice: a protocol for an implementation study. BMJ Open. 2023;13(3):e068603. 36863741 10.1136/bmjopen-2022-068603 PMC9990614

[11] Boudewijns EA, Claessens D, van Schayck OCP, Twellaar M, Winkens B, Joore MA, et al. Effectiveness of the Assessment of Burden of Chronic conditions (ABCC)-tool in patients with asthma, COPD, type 2 diabetes mellitus, and heart failure: a pragmatic clustered quasi-experimental study in the Netherlands. Eur J Gen Pract. 2024;30(1):2343364. 38738695 10.1080/13814788.2024.2343364 PMC11104697

[12] Claessens D, Vervloet M, Boudewijns EA, Gidding-Slok AHM, van Schayck OCP, van Dijk L. Barriers and facilitators to the implementation of the Assessment of Burden of Chronic Conditions tool in Dutch primary care – a context analysis (manuscript submitted). 2024. 10.1186/s12913-024-11270-y PMC11264986 39033106

[13] Tsiachristas A, Dikkers C, Boland MR, Rutten-van Molken MP. Exploring payment schemes used to promote integrated chronic care in Europe. Health Policy. 2013;113(3):296–304. 23937868 10.1016/j.healthpol.2013.07.007

[14] Forman J, Heisler M, Damschroder LJ, Kaselitz E, Kerr EA. Development and application of the RE-AIM QuEST mixed methods framework for program evaluation. Prev Med Rep. 2017;6:322–8. 28451518 10.1016/j.pmedr.2017.04.002 PMC5402634

[15] Glasgow RE, Harden SM, Gaglio B, Rabin B, Smith ML, Porter GC, et al. RE-AIM planning and evaluation Framework: adapting to New Science and Practice with a 20-Year review. Front Public Health. 2019;7:64. 30984733 10.3389/fpubh.2019.00064 PMC6450067

[16] Glasgow RE, Vogt TM, Boles SM. Evaluating the public health impact of health promotion interventions: the RE-AIM framework. Am J Public Health. 1999;89(9):1322–7. 10474547 10.2105/AJPH.89.9.1322 PMC1508772

[17] Carroll C, Patterson M, Wood S, Booth A, Rick J, Balain S. A conceptual framework for implementation fidelity. Implement Sci. 2007;2:40. 18053122 10.1186/1748-5908-2-40 PMC2213686

[18] Slok AH, Twellaar M, Jutbo L, Kotz D, Chavannes NH, Holverda S, et al. To use or not to use’: a qualitative study to evaluate experiences of healthcare providers and patients with the assessment of burden of COPD (ABC) tool. NPJ Prim Care Respir Med. 2016;26:16074. 27853139 10.1038/npjpcrm.2016.74 PMC5113148

[19] van Leersum CM, Moser A, van Steenkiste B, Wolf J, van der Weijden T. Clients and professionals elicit long-term care preferences by using ‘What matters to me’: a process evaluation in the Netherlands. Health Soc Care Community. 2022;30(4):e1037–47. 34254385 10.1111/hsc.13509 PMC9291068

[20] Hogervorst S, Adriaanse M, Brandt H, Vervloet M, van Dijk L, Hugtenburg J. Feasibility study of a digitalized nurse practitioner-led intervention to improve medication adherence in type 2 diabetes patients in Dutch primary care. Pilot Feasibility Stud. 2021;7(1):152. 34362471 10.1186/s40814-021-00892-2 PMC8349070

[21] Beentjes TAA, van Gaal BGI, Vermeulen H, Nijhuis-van der Sanden MWG, Goossens PJJ. A blended electronic illness management and recovery program for people with severe Mental illness: qualitative process evaluation alongside a Randomized Controlled Trial. JMIR Ment Health. 2021;8(1):e20860. 33470945 10.2196/20860 PMC7857951

[22] Sieverink F, Kelders S, Braakman-Jansen A, van Gemert-Pijnen J. Evaluating the implementation of a personal health record for chronic primary and secondary care: a mixed methods approach. BMC Med Inf Decis Mak. 2019;19(1):241. 10.1186/s12911-019-0969-7 PMC6882368 31775734

[23] Versluis A, van Luenen S, Meijer E, Honkoop PJ, Pinnock H, Mohr DC, et al. SERIES: eHealth in primary care. Part 4: addressing the challenges of implementation. Eur J Gen Pract. 2020;26(1):140–5. 33025820 10.1080/13814788.2020.1826431 PMC7580793

[24] Alpay LL, Henkemans OB, Otten W, Rovekamp TA, Dumay AC. E-health applications and services for patient empowerment: directions for best practices in the Netherlands. Telemed J E Health. 2010;16(7):787–91. 20815745 10.1089/tmj.2009.0156

[25] Vainauskiene V, Vaitkiene R. Enablers of patient knowledge empowerment for self-management of Chronic Disease: an integrative review. Int J Environ Res Public Health. 2021;18(5). 10.3390/ijerph18052247 PMC7956493 33668329

[26] Damschroder LJ, Reardon CM, Widerquist MAO, Lowery J. The updated Consolidated Framework for Implementation Research based on user feedback. Implement Sci. 2022;17(1):75. 36309746 10.1186/s13012-022-01245-0 PMC9617234

[27] Voorhaar M, van Schayck OCP, Winkens B, Muris JWM, Slok AHM. It is Smart to set treatment goals, but are Set Treatment Goals SMART? A qualitative Assessment of goals described in the Assessment of the Burden of COPD Tool. COPD. 2023;20(1):357–62. 38178806 10.1080/15412555.2023.2289908

[28] van Dijk-de Vries A, van Bokhoven MA, de Jong S, Metsemakers JF, Verhaak PF, van der Weijden T, et al. Patients’ readiness to receive psychosocial care during nurse-led routine diabetes consultations in primary care: a mixed methods study. Int J Nurs Stud. 2016;63:58–64. 27597730 10.1016/j.ijnurstu.2016.08.018

[29] Powell BJ, Beidas RS, Lewis CC, Aarons GA, McMillen JC, Proctor EK, et al. Methods to improve the selection and tailoring of implementation strategies. J Behav Health Serv Res. 2017;44(2):177–94. 26289563 10.1007/s11414-015-9475-6 PMC4761530

[30] Baker R, Camosso-Stefinovic J, Gillies C, Shaw EJ, Cheater F, Flottorp S, et al. Tailored interventions to address determinants of practice. Cochrane Database Syst Rev. 2015;2015(4):CD005470. 25923419 10.1002/14651858.CD005470.pub3 PMC7271646

[31] Slok AH, in ‘t Veen JC, Chavannes NH, van der Molen T, Rutten-van Molken MP, Kerstjens HA, et al. Development of the Assessment of Burden of COPD tool: an integrated tool to measure the burden of COPD. NPJ Prim Care Respir Med. 2014;24:14021. 25010353 10.1038/npjpcrm.2014.21 PMC4498164

