# Supplementary material for: Process evaluation of the implementation of the assessment of burden of chronic conditions tool in Dutch primary care – lessons from a qualitative implementation study
Source: BMC Health Serv Res. 2024 Jul 20;24:827. doi: 10.1186/s12913-024-11270-y (PMC11264986; doi:10.1186/s12913-024-11270-y)
Supplement: Supplementary file 2 — Supplementary Material 2 [file 12913_2024_11270_MOESM2_ESM.docx]

**The Assessment of Burden of Chronic Conditions (ABCC-)tool**

Description of the tool

The Assessment of Burden of Chronic Conditions (ABCC-)tool has been developed as a structured instrument to guide the conversation between healthcare provider (HCP) and people with chronic conditions towards personalized care plans. The tool aims to assess a patient’s experienced burden, visualize the results during the conversation, and facilitate the formulation of personalized care goals and plans. The tool consists of three main practical components: 1) a questionnaire assessing burden, 2) a visualization of the results of the questionnaire, and 3) treatment advice to facilitate personalized care plans, including goals. **The questionnaire** is developed to assess the experienced burden for people with either asthma, Chronic Obstructive Pulmonary Disease (COPD), type 2 diabetes mellitus (T2DM) or Chronic Heart Failure (CHF). To allow the ABCC-tool to be applied to multiple chronic conditions, it has a modular design, consisting of a generic set of questions that are applicable to any chronic conditions and disease-specific additional sets of questions. These sets of questions are combined into a single questionnaire that is appropriate for the patient. The generic questionnaire consists of ten items clustered into seven domains: physical limitations, fatigue, night’s rest, feelings/emotions, sexuality, and medicines. Additionally, lifestyle questions assess someone’s behavior regarding bodyweight or Body Mass Index, physical activity, smoking or alcohol consumption. For someone with COPD, six additional questions are represented in two additional domains (pulmonary complaints and lung attacks). For someone with asthma, seven additional questions are represented in three additional domains (asthma complaints, lung attacks, and nasal complaints). For someone with T2DM, four additional questions are represented in four additional domains. For someone with CHF nine additional questions are represented in four additional domains. All questions are scored on a 7-point Likert scale from no burden ( score 0) to highest burden (score 6). In addition, an open-ended question allows the patient to ask questions or provide additional relevant discussion topics. The **visualization** presents the results of the questionnaire as a balloon chart, with each domain represented by a balloon. These balloons range from highest height and green color to represent no burden, to lowest height and red color to represent the most burden. Balloons decline and turn orange and yellow with increasing burden. Additionally, the results from the previous visit are depicted as grey to allow for monitoring of progress. In addition to balloons, the input from the open-ended question is presented below the balloon chart. Upon clicking on a balloon, **treatment advice** is presented in accordance to disease-specific guidelines from the Dutch College of General Practitioners (*Nederlands Huisartsgenootschap, NHG, in Dutch*). A set of options is presented for both HCP and patient to discuss and select the most appropriate. These options are aimed at guiding the formulation of personalized care plans, including goals.

Intended practical use of the tool

The cycle of using the ABCC-tool contains several steps (see figure 1 op additional file 1). **First**, a patient completes the questionnaire of the ABCC-tool prior to the consultation. **Second**, the outcomes of the questionnaire are digitally transformed to a balloon chart. This balloon chart is intended to be presented at the start of the conversation. Patient and HCP review the balloon chart together and discuss which domains or topics are most relevant to discuss. **Third**, HCP and patient select one or a few topics from the balloon chart to an in-depth clarification of the difficulties the patient experiences with regard to those domains. HCPs are instructed to apply the principles of shared decision-making to assist the patient in selecting relevant topics. The choice of domains is not further influenced and left to the shared decision of HCP and patient. **Fourth**, after clarifying the patient’s experience and difficulties, the patient should be invited to identify a healthcare-related goal and formulate a personalized care plan, using motivational interviewing. To assist the patient in formulating care goals and plans, the HCP should provide the patient with options that can be based on the treatment advice from the ABCC-tool. The benefits and drawbacks of each option should be discussed in order for the HCP to elicit preferences from the patient. At the end of this step, a specific and personalized care goal and plan should be formulated. HCPs are instructed that they can use the Specific-Measurable-Acceptable-Realistic-Timely (SMART) principles for structuring these goals. **Fifth**, patients should be monitored using the ABCC-tool during their routine visits. Monitoring of progress should include discussing the progress on the care goal and the change in experienced burden as depicted by the combination of colored and grey balloons.

*
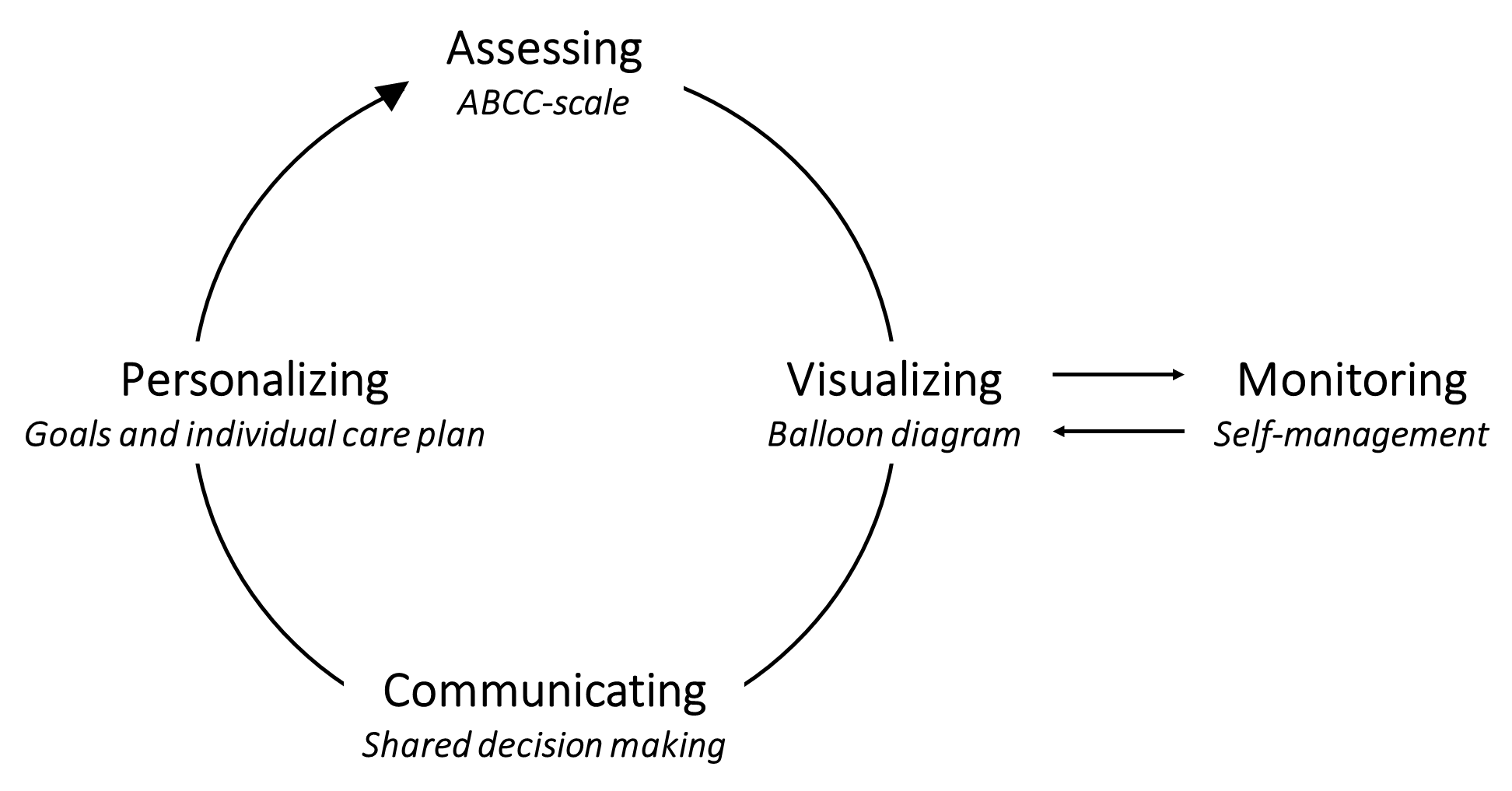
*

Figure 1: Cycle of using the ABCC-tool

Intended position of ABCC-tool within the clinical consultation

The ABCC-tool requires a specific moment of use within the clinical consultation for it to be able to effectively facilitate shared-decision making while minimizing its time demands. The ABCC-tool should exclusively be used as the start of the clinical consultation. The tool is developed to measure experienced burden of disease before the consultation, and facilitate a shared choice on discussion topics during the consultation. Initiating the conversation using the ABCC-tool’s visualization is therefore essential to its potential benefit. Any discussion topics that fell outside of the scope of the tool should be discussed after formulating personal care goals (i.e. completing the fourth step of the ABCC-tool cycle).

Contents of practical information provided to participating HCPs.

Participants were provided practical information about how to use the ABCC-tool **in paper**. The first part of the information contained a simplified version of the description presented above. The second part was a step-by-step description of the cycle of using the ABCC-tool, including examples of possible domains of burden and potential goals that can be formulated in response of these domains. This step-by-step approach has been translated into an office-ready explanation poster that served as a practical reminder of the steps of the ABCC-tool. The third part of the practical information contained the background information of how the modules of the ABCC-tool (generic, asthma, COPD, T2DM) were combined into a single questionnaire and visualization. HCPs received a set of paper copies of all single conditions and possible combinations (e.g. asthma-DM2, COPD-DM2) which were ready to be distributed among patients. HCPs also received digital versions to be used per e-mail. The fourth part was a step-by-step explanation of how to find and use the ABCC-tool in their information system. Additionally, HCPs received a **video-explainer** of all these steps via e-mail along the practical information on paper. The practical information and sets of questionnaires were distributed in person by one of the researchers in an in-person appointment where HCPs were provided the opportunity to ask questions.

In summary, HCPs received practical information on paper and video containing:

1. A description of the ABCC-tool
2. A step-by-step explanation of the cycle of using the ABCC-tool
3. Background information about the construction of the questionnaire, including prepared paper versions for all conditions and possible combinations
4. A step-by-step guide of how to find and use the ABCC-tool within their information system
