# Supplementary material for: Process evaluation of the implementation of the assessment of burden of chronic conditions tool in Dutch primary care – lessons from a qualitative implementation study
Source: BMC Health Serv Res. 2024 Jul 20;24:827. doi: 10.1186/s12913-024-11270-y (PMC11264986; doi:10.1186/s12913-024-11270-y)
Supplement: Supplementary file 3 — Supplementary Material 3 [file 12913_2024_11270_MOESM3_ESM.docx]

**Additional file 3: Interview guide to the process evaluation of the ABCC-tool**

1. **How have you experienced working with the ABCC-tool?**

From our previous interview, we discussed that several factors were of **positive** influence to you using the ABCC-tool in practice. These were:

1. <to be included from previous interview>
2. <to be included from previous interview>
3. **Did you experience the influence of any of these in your daily practice?**
   1. How did this effect you using the ABCC-tool?

From our previous interview, we discussed that several factors were of **negative** influence to you using the ABCC-tool in practice. These were:

1. <to be included from previous interview>
2. <to be included from previous interview>
3. **Did you experience the influence of any of these in your daily practice?**
   1. How did this effect you using the ABCC-tool?

Using the ABCC-tool was prescribed as following several steps. We would like to know more about each of these steps in detail. In short, the steps of using the ABCC-tool were: assessing experienced burden using the questionnaire, visualizing the results using the balloon chart, involving the patient in the conversation about the balloon chart through the application of shared decision making, formulating personalized care plans including goals and monitoring progress.

1. **To what extent did you follow these steps?**
   1. Could you express how you felt about these steps?
   2. Which steps were most useful to you?
   3. Why were these steps more useful than other?
   4. Why were other steps less useful to you?
   5. Did you experience the need to apply the ABCC-tool differently among patients?
2. **How exactly did you use the ABCC-tool in daily practice?**
   1. How did you use the questionnaire of the ABCC-tool?
   2. How did you use the visualization of the ABCC-tool?
   3. How did you involve the patient in your conversation?
   4. How did you formulate care plans and goals?
   5. How did you monitor the patient’s progress?
3. **How much time did you and your patients need to use the ABCC-tool?**
   1. What is your opinion about the amount of time you needed?
   2. How would you evaluate the amount of time spent on the ABCC-tool with respect to the entire consultation?
   3. Was the time you spent on the ABCC-tool worth it to you? Why?
4. **How would you describe the people you used the ABCC-tool with?**
   1. What were reasons for your patients to participate or decline?
   2. Did you use the ABCC-tool with patients that did not participate in the effectiveness trial? If so, please elaborate on their characteristics and your reasons to use the ABCC-tool with these patients?
5. **Did you notice any effect on patients from using the ABCC-tool?**

*If no response, explain: You could think of how they experience their care or quality of life, or even how active they participate in the conversation.*

- 1. How would you describe this effect?
  2. What role did the ABCC-tool play in this effect?

We would also like to know how you view using the ABCC-tool after the study period.

1. **How would you use the ABCC-tool after finishing the study period?**
   1. Do you expect to use the ABCC-tool similarly after the study period? Why, and how would you change it if not?
   2. Which healthcare providers do you expect to use the ABCC-tool? Why?
   3. With whom do you expect to use the ABCC-tool with in practice? What characterizes these patients? *If no response, explain: you can think of different types of chronic conditions, but also age, motivation etc.*
   4. How would using the ABCC-tool fit with current working structures and time availability?
   5. Did you make changes to using the ABCC-tool, or are they required, to continue using the ABCC-tool in your practice? *If no response, explain: You can think of changes to the ABCC-tool itself, but also structural, technical or financial changes in your practice*
   6. Are you willing to make these changes to using the ABCC-tool or your practice to continue using the ABCC-tool?
2. **Is any form of training required to be able to use the ABCC-tool?**

**If yes:**

- 1. What should definitely be part of this training in your opinion?
  2. To whom should this training be offered in your opinion?
  3. Who would be providing these trainings in your opinion?

**If no:**

- 1. What other methods could we apply to assist healthcare providers in getting acquainted with the ABCC-tool?

1. **Would you recommend using the ABCC-tool to your colleagues?**

**If yes:**

- 1. To whom would you recommend using the ABCC-tool? *If no clear response, explain: general practitioners, nurses, other healthcare providers?*
  2. Why would you recommend it to them?

**If no:**

- 1. Why would you not recommend using the ABCC-tool?
